# Evolution and subfamilies of HERVL human endogenous retrovirus

**DOI:** 10.1093/bioadv/vbae110

**Published:** 2024-07-30

**Authors:** Huan Zhang, Martin C Frith

**Affiliations:** Department of Computational Biology and Medical Sciences, University of Tokyo, Chiba 277-8561, Japan; Department of Computational Biology and Medical Sciences, University of Tokyo, Chiba 277-8561, Japan; Artificial Intelligence Research Center, AIST, Tokyo 135-0064, Japan; Computational Bio Big-Data Open Innovation Laboratory, AIST, Tokyo 169-8555, Japan

## Abstract

**Background:**

Endogenous retroviruses (ERVs), which blur the boundary between virus and transposable element, are genetic material derived from retroviruses and have important implications for evolution. This study examines the diversity and evolution of human endogenous retroviruses (HERVs) of the HERVL family, which has long terminal repeats (LTRs) named MLT2.

**Results:**

By probability-based sequence comparison, we uncover systematic annotation errors that conceal the true complexity and diversity of transposable elements (TEs) in the human genome. Our analysis identifies new subfamilies within the MLT2 group, proposes a refined classification scheme, and constructs new consensus sequences. We present an evolutionary analysis including phylogenetic trees that elucidate the relationships between these subfamilies and their contributions to human evolution. The results underscore the significance of accurate TE annotation in understanding genome evolution, highlighting the potential for misclassified TEs to impact interpretations of genomic studies.

**Availability and implementation:**

Not applicable.

## 1 Introduction

Transposable elements (TEs) are abundant mobile genetic elements that can move or copy themselves within the genome: they play a pivotal role in its evolution, gene regulation, and chromosomal rearrangements. Their relics account for about half of the human genome. In this study, we focus on endogenous retroviruses (ERVs): they are a type of TE, and they are genetic material derived from retroviruses.

Among retrotransposons, there are two significant subclasses: Non-long terminal repeat (LTR) retrotransposons, which include LINEs and SINEs, and LTR retrotransposons (Kojima 2019). The latter subclass includes ERVs. ERVs, derived from exogenous retroviruses, have the same (duplicated) DNA sequence at their left and right edge: these are termed LTRs, and internal regions containing three genes: gag, pol, and env (Ueda *et al.* 2020). Mammal genomes contain many solo LTRs, which presumably arose by homologous recombination between the two LTRs of an ERV.

ERVs are derived from ancient retroviral infections and can either proliferate through infection of germline cells like typical retroviruses or behave as retrotransposons when they lack the env gene necessary for cell entry (Magiorkinis *et al.* 2012).

ERVs have important implications for evolution, as they can promote the production of new genes, regulate gene expression, and participate in placental formation and immune responses (Johnson 2019). ERVs are also associated with a number of diseases, such as cancer and neurodegenerative diseases (Römer 2021).

ERVs provide a unique perspective on the co-evolution of viruses and their hosts, as they carry molecular signatures of historical infections. While most ERVs are no longer replication-competent, having accumulated numerous mutations and deletions over millions of years, some retain the ability to produce functional proteins and even virus-like particles, particularly those from the more recent HERVK(HML2) family (Vargiu *et al.* 2016). These elements can still impact host genomes by providing alternative regulatory elements like promoters and enhancers, contributing to the complexity of gene regulation.

The impact of ERVs extends beyond simple remnants of past infections; they are integral to the host’s evolutionary adaptation, providing raw genetic material that can be co-opted for new functions. For instance, the ERV-derived gene syncytin has been repurposed during mammalian evolution for a role in placental development (Ueda *et al.* 2020). This illustrates how TEs, particularly ERVs, are not just passive elements but active participants in genomic innovation and adaptation.

However, classifying ERV lineages within current taxonomic systems presents considerable challenges. Due to the high degree of similarity among transposable elements, many ERVs have been assigned somewhat arbitrary names within repetitive element classification systems, complicating the accurate categorization of these genetic elements (Gifford *et al.* 2018). Furthermore, the high similarity among transposable elements often makes the classification process particularly difficult, leading to issues in accurately annotating these elements within the genome.

MLT2 is a prominent family of LTRs with several subfamilies, harboring about 35 000 copies per haploid human genome. Originating from HERVL ERVs within the ERV3 class, these retroviruses have had a substantial impact on the genome. They have facilitated the retrotransposition of non-autonomous MaLR (Mammalian apparent LTR-retrotransposon) elements, making MaLRs the most abundant LTR-type repeat, with approximately 386 000 copies per haploid genome (Smit 1993, Franke *et al.* 2017).

In this study, we show that annotations of TEs in genomes have a kind of systematic error that hides TE diversity and complexity. We observed that subfamilies within the MLT2 family exhibited incomplete classification, leading to inaccuracies in genomic annotation. We identified new subfamilies within MLT2 in the human genome and constructed new consensus sequences. Also, we analyzed the evolutionary relationships between these subfamilies and reconsidered their functions. This approach aims to enhance the understanding of the evolutionary dynamics and functional implications of MLT2 and its subfamilies, contributing to more precise genomic annotations and insights into the evolutionary history of these significant genetic elements.

## 2 Materials and methods

### 2.1 Analysis of interspersed repeats in the human genome

We first downloaded consensus DNA sequences of human repeats from Dfam version 3.7, a database of transposable element families (Storer *et al.* 2021). We then annotated repeats in the human genome (GRCh38/hg38), by comparing the genome to these consensus sequences using LAST version 1411 (https://gitlab.com/mcfrith/last). The key advantage of LAST is its resolution of ambiguity, where one part of the genome is similar to more than one repeat. LAST optimizes division of the genome into parts based on alignment probabilities of the parts to different repeats (Frith and Kawaguchi 2015). After this procedure, each genome base-pair is aligned to at most one Dfam base-pair. Thus, it can detect “hybrid TEs”, that is, two or more TEs that appear in the genome as hybrids due to homologous recombination or other reasons and are usually recognized as a single TE (Carey *et al.* 2021a). last-train was used to determine the average rates of insertion, deletion and substitutions between genome and repeat consensus sequences. The substitution rates form a 4 × 4 matrix, shown in the supplement. Note that some alternative alignment tools, such as NCBI BLAST, do not allow such a custom 4 × 4 matrix. These rates of substitution, deletion, and insertion were used to calculate alignment probabilities, and the most likely division of the genome into parts.

Finally, we used TE-reX (Pascarella *et al.* 2022), a tool that builds upon the results produced by LAST, to identify and extract “hybrid TEs” from the genome. Related details and additional information can be found on our GitHub repository: https://github.com/zh1996-jp/MLT2-classification.

### 2.2 Classification of subfamilies

To refine the classification of subfamilies, we began with self-comparisons of targeted subfamily instances using rmblast.pl, in RepeatModeler version 2.0.3 (Flynn *et al.* 2020) using the -ms 2000 command to set a high minimum score threshold. The highest-scoring instance from this comparison was selected as the consensus sequence. This consensus sequence was then input into alignAndCallConsensus.pl, where it was compared against other instances. This program enables the consensus sequence to be modified based on the results of the multiple sequence alignment. The results from alignAndCallConsensus.pl were visualized using the -html option and stored in the con.ali file. The GrepCrossmatch tool was used to identify sequences with similar features from the con.ali file. If a significant number of sequences differed from the consensus and shared distinct features, indicating potential new subfamilies, they were extracted using getSeqfromXMlines.pl for the establishment of new subfamilies.

The above processes were repeated until no new subfamilies were detected.

Finally, rmblast.pl was used again to perform self-comparisons on the newly established subfamilies, with command -ms 2000 to ensure high minimum score, and -masklevel 101 to return all matches over the cutoff score, not only the best. This step was to ensure the differences between subfamilies.

### 2.3 Construction of evolutionary tree

We extracted from the genome MLT2 instances larger than 300 base pairs that start with TG and end with CA (the terminal dinucleotides of ERVs), and aligned them using MAFFT version 7 (Katoh and Standley 2013). We employed the command mafft -auto, which allows MAFFT to select the optimal alignment strategy for our data. To enhance the reliability of these alignments, we used the tool trimAl (Capella-Gutiérrez *et al.* 2009) with the option -gt 0.01. This parameter setting ensures the exclusion of less reliable parts of the alignment by retaining only those columns in the alignment where at least 1% of the sequences contain a non-gap character.

The resulting aligned sequences were then input into FastTree (Price *et al.* 2010), which efficiently generates maximum-likelihood trees for large datasets, with -nt to indicate that the input sequences are nucleotides. The final phylogenetic tree was visualized using chiplot (Xie *et al.* 2023).

### 2.4 Evolutionary age and conservation analysis

We employed lift-over (Kent *et al.* 2002) to investigate genomic similarities across species. This tool was used to identify and quantify new family sequences that are shared between the human genome and other species’ genomes. By mapping these sequences from one species’ genome to another, lift-over helps in understanding the evolution and conservation of genetic elements across different organisms.

## 3 Results and discussion

### 3.1 Discovery of a new MLT2 subfamily

**Figure 1.**
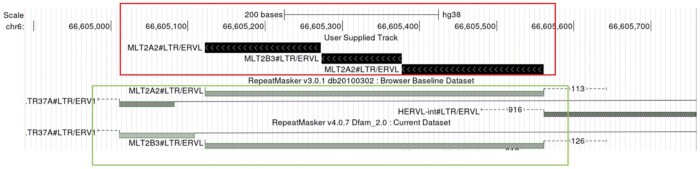
Screen shot of human chromosome 6: 66605123–66607729. The annotation of hybrid TE aba. In the upper red box is the annotation of LAST, which shows that the element consists of three parts. In the lower green box is the annotation of RepeatMasker, whose two versions annotate the element as a single MLT2B3 and MLT2A2, respectively.

By comparing the human genome to repeat consensus sequences, we detected 36,466 hybrid elements (as explained in Section 2) in the genome, and discovered an unusual type of hybrid LTR, which we named “aba”. It consists of three parts, with the sequence at both ends matching the MLT2A2 LTR family, and the middle segment matching MLT2B3 (Fig. 1). Both elements are from the MLT2 group of LTRs, in the ERV3 class.

This hybrid TE appears more than 2000 times in the human genome, with roughly fixed “breakpoints”. The “breakpoint” indicates the position in the consensus sequence at which an element is replaced by another element. The phylogenetic tree also shows that the aba sequence is distinct from other sequences in the MLT2 family. Based on these results we infer that a new subfamily can be separated from MLT2A2 and MLT2B3.

### 3.2 Reclassification of subfamilies

We first aligned the extracted instances of these families with the consensus sequence for multiple sequence alignment (MSA). We posited that sequences capable of segregating from others to form a new subfamily must exhibit distinct characteristics not found in the rest of the sequences. For instance, when aligned with the consensus sequence of their current subfamily, these sequences consistently ceased to match at a specific position along the consensus sequence. This behavior—a hallmark of sequences with a fixed pattern and a defined stopping point—was used as a criterion to extract and classify these sequences into new subfamilies.

**Figure 2.**
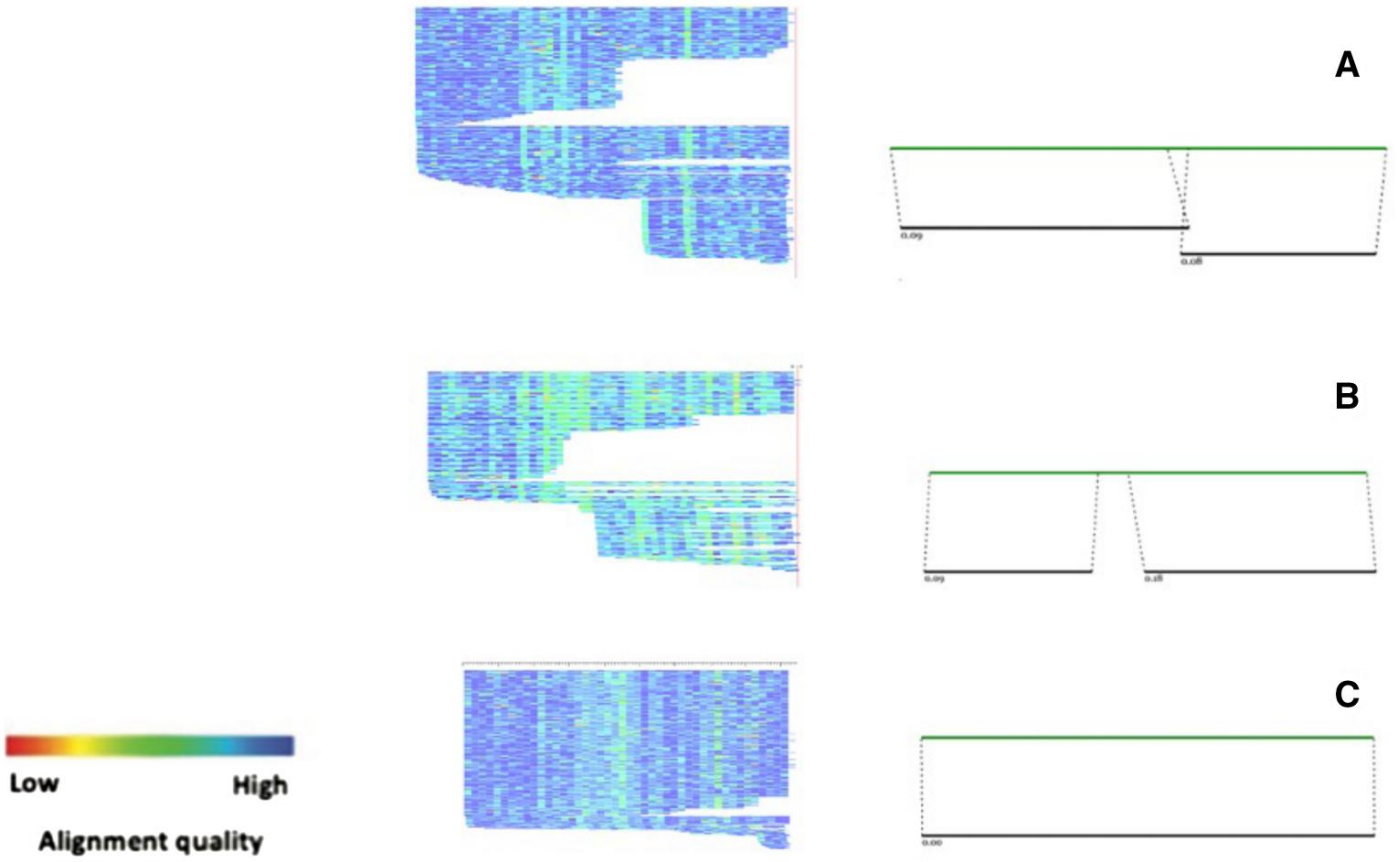
Alignments of consensus sequences to LTR instances, for three LTR groups. The alignments were generated using the alignAndCallConsensus.pl script from RepeatModeler, The top horizontal line of each graph is the consensus sequence, and the lines below it are LTR instances. The color gradient represents the quality of alignment, where darker shades indicate higher alignment quality and lighter shades indicate lower quality or mismatches. Group A displays the MSA results for aba. In the left image of Group A, we can observe that a large number of instance sequences do not align well with the consensus sequence in the middle section. This is specifically illustrated in the right image, where we can see a gap where the alignment does not match. Group B presents the MSA results for MLT2B3, which, similar to aba, show a majority of sequences with unaligned middle segments, indicated by a gap in the right image. Group C shows the MSA results for MLT2A2, which markedly differ from the previous two groups, as detailed in the right image. Most sequences align well with the consensus sequence across the entire length, indicating no further subfamily separation is required.

Subsequently, from the MSA results of aba and MLT2B3, we identified subfamilies that could be separated from each group (Fig. 2). Within both subfamilies, sequences exhibited a distinctive pattern by ceasing to align at fixed positions (For further details, refer to Supplementary Figs S1 and S2). We named the subfamilies extracted from aba and MLT2B3 as subaba and subB3, respectively. Consequently, we expanded the classification of the original MLT2A2 and MLT2B3 subfamilies into five distinct groups: MLT2A2, MLT2B3, subB3, aba, and subaba.

### 3.3 Differences between subfamilies

We checked that there are differences between the obtained subfamilies, using rmblast.pl to compare the consensus sequences of each subfamily, keeping only the results with scores > 2000. The lengths of MLT2A2, MLT2B3, subB3, aba, and subaba are 453, 550, 421, 554, and 519 bp respectively. According to rmblast.pl results: subaba has 7.13% deletion/insertion relative to aba, and 14.57% relative to MLT2A2. In contrast, aba is similar to MLT2A2 but still has deletion/insertion of 22.74%, and from MLT2B3 rmblast.pl reports a divergence of 9.39%, and deletion/insertion of about 6%. subB3 has a divergence of 4.51% and 5% deletion/insertion compared to MLT2B3.

**Figure 3.**
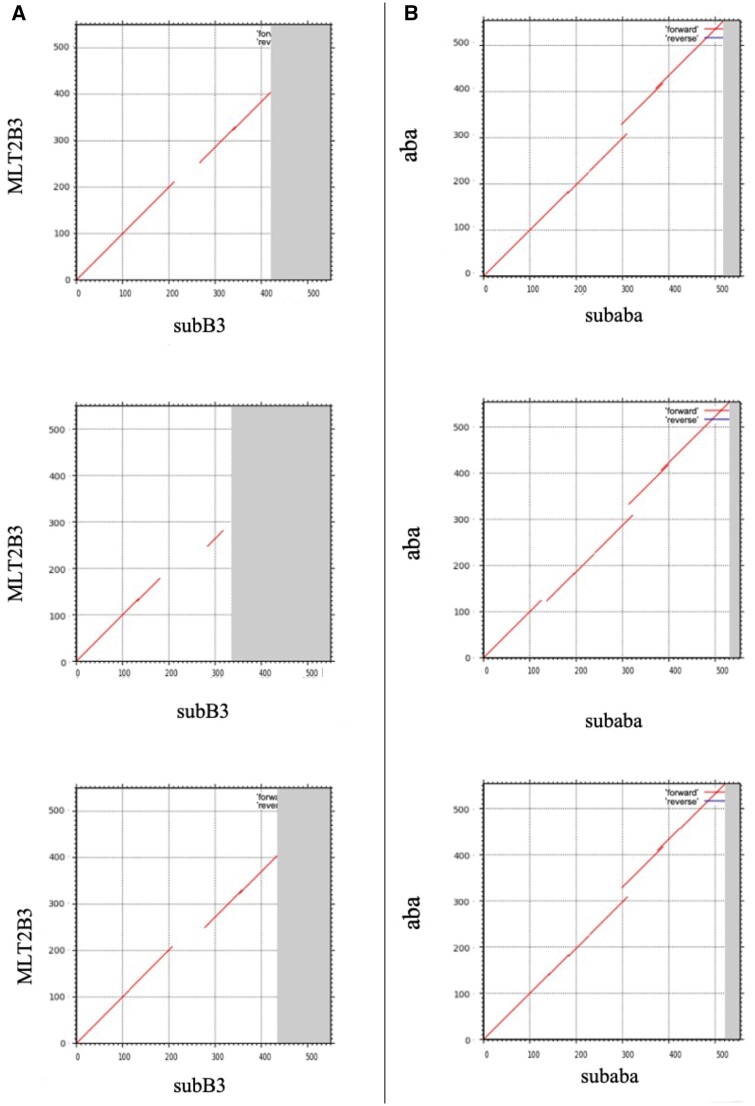
Panel (A) shows sequence alignment of the newly constructed consensus sequence of MLT2B3 with three randomly selected subB3 instances. Panel (B) shows aba aligned to instances of subaba. We can see consistent differences between MLT2B3 and subB3, aba and subaba. The horizontal coordinates represent each instance while the vertical coordinates represent consensus sequences of MLT2B3 and aba.

Subsequently, we used MAFFT to align the consensus sequences of MLT2B3 and aba with instances of subB3 and subaba, respectively. This shows that the MLT2B3 sequence has a region not similar to subB3 at 200-250bp, and aba has a consistent insertion relative to subaba at 300 bp (Fig. 3).

**Figure 4.**
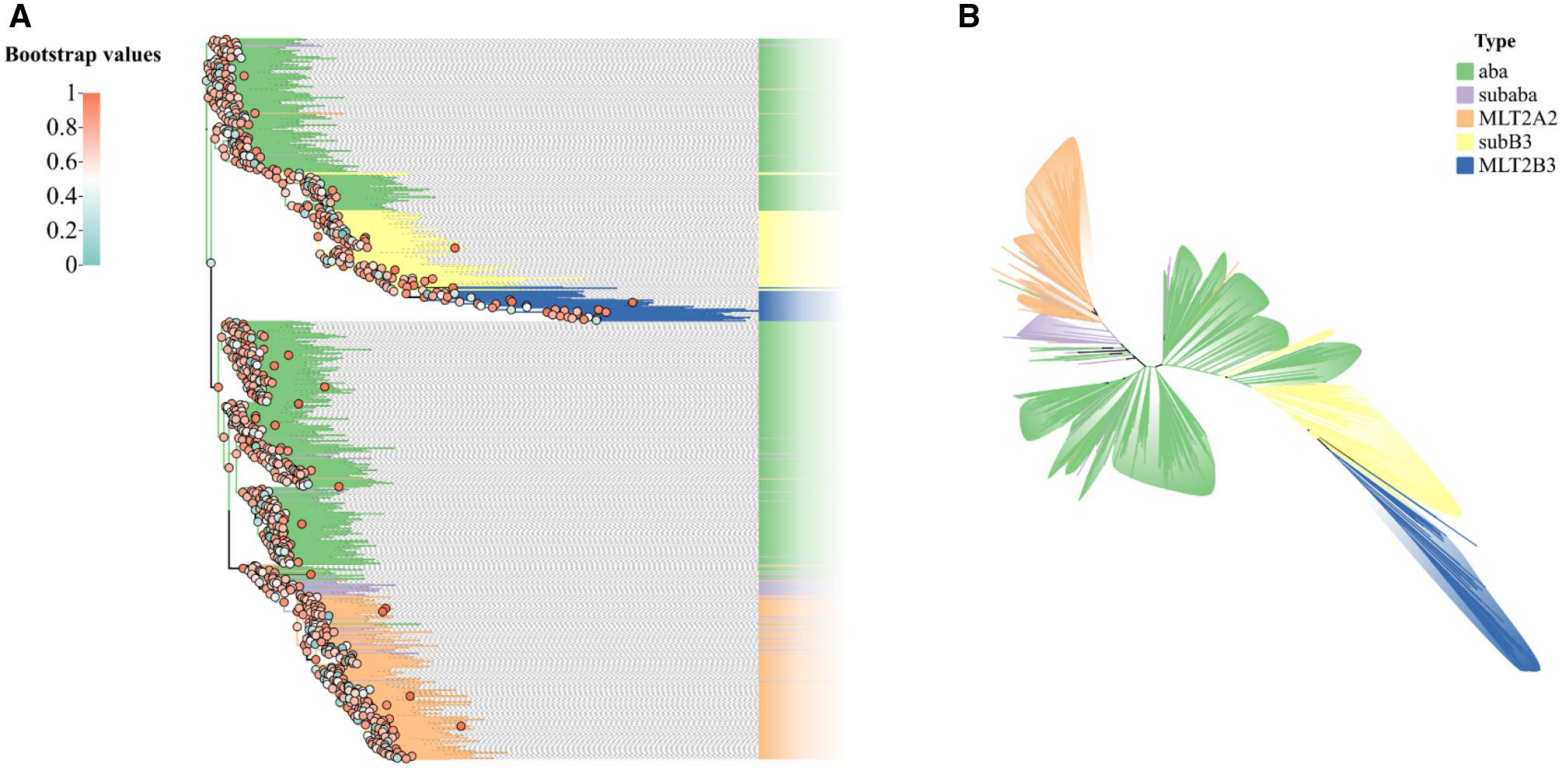
Evolutionary tree constructed with new subfamily sequences. Figure (A) represents a rooted phylogenetic tree constructed using sequences from the newly established subfamilies. Bootstrap values are indicated by colored circles, illustrating the statistical support for each node. Sequences from the same family are well-clustered together, signifying their close evolutionary relationships. Figure (B) depicts an unrooted tree, It can be observed that aba appears to be an intermediary, with the evolutionary trajectories of other families seemingly radiating out from it.

The evolutionary tree (Fig. 4) reflected the differences between the newly created subfamilies. aba sequences were only slightly mixed with MLT2A2 sequences, and subB3 with MLT2B3 sequences in the tree. As stated in the Methods, the sequences used for tree building are all DNA segments >300 bp and of the form TG…CA.

### 3.4 Evolutionary age of the subfamilies

**Figure 5.**
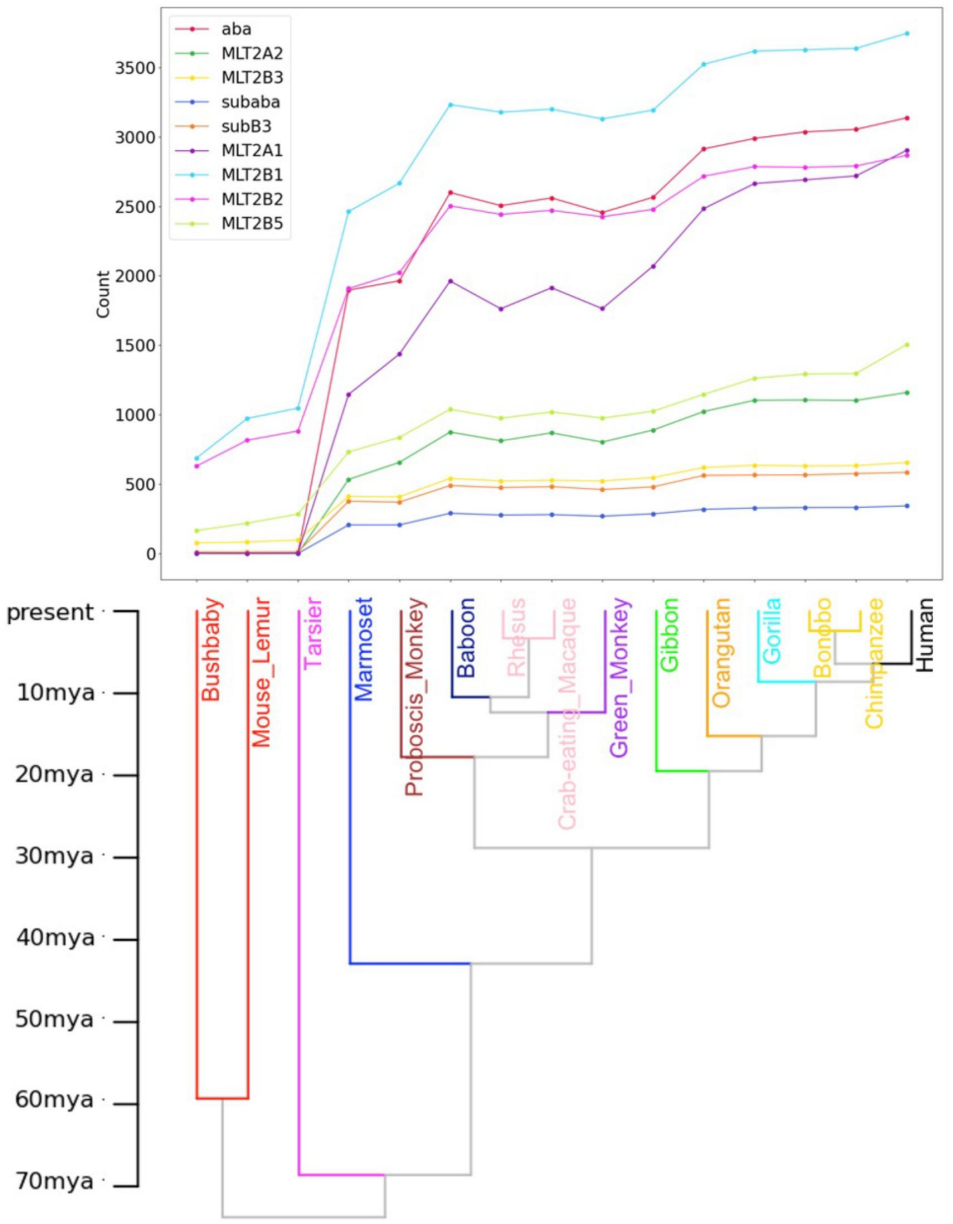
Evolutionary age of MLT2 subfamilies. The horizontal coordinates are arranged according to the estimated age of the different species’ divergence from human, from about 60 mya ago to present. The vertical coordinate is the number of new family sequences shared by human and other genomes.

We used liftover to project sequences of new families in the human genome to different genomes, and then counted the number of new family sequences shared by human and other genomes. MLT2B3 has the oldest insertion age of all new subfamilies, dating back to the human/bushbaby common ancestors, and aba is the fastest growing subfamily since human/tarsier common ancestors. Most of these DNA elements were inserted before the last common ancestor of all simians, and after their common ancestor with tarsiers (Fig. 5). We can speculate that they played a major role in the evolution of simians.

### 3.5 The new subfamilies contributed to human evolution

A previous study (Hashimoto *et al.* 2021) showed that MLT2A elements can act as promoters in early embryonic development and contribute to the regulation of gene expression in somatic tissues. They found that more than 300 of these elements retain promoters that are functional in four- or eight-cell blastomeres: 81 of them are MLT2A2 elements. The authors also discovered that 21 MLT2A elements are expressed in human pineal gland or amniotic membrane. Three of them serve as novel promoters of protein-coding genes ABCE1, COL5A1, and GALNT13, in the pineal gland of humans but not in that of macaques.

We found that among the 81 MLT2A2 elements expressed in four- or eight-cell blastomeres, there were actually 25 aba, 4 MLT2A1, 43 MLT2A2, and 9 subaba elements. Meanwhile, although the overall expression levels (TPM values) were small, the two elements with TPM >50 were subaba and MLT2A1.

Among the 21 MLT2A elements that are expressed in human pineal gland or amniotic membrane, 6 were reported as MLT2A2. We found that 5 are aba and 1 is subaba. Moreover, among the 3 MLT2A elements that can act as promoters, the GALNT13 promoter was reported as MLT2A2: we found it to be aba.

Thus, accurate recognition of ERV subfamilies clarifies the intertwined stories of ERV and human evolution.

**Figure 6.**
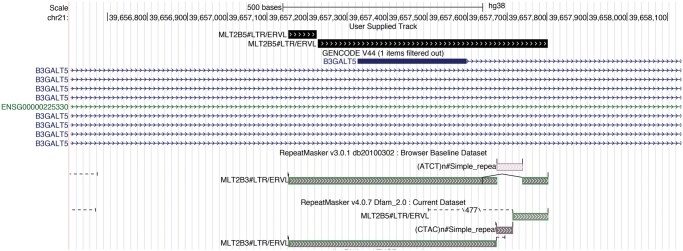
Screenshot of a promoter and first exon of B3GALT5. “User Supplied Track” is the LAST result which annotates this region with MLT2B5. In the RepeatMasker results, v3.0.1 annotates it as a full MLT2B3; however, v4.0.7 annotates it as partly MLT2B3 and partly MLT2B5.

Likewise, MLT2B3 has been implicated in a variety of functional roles. However, due to imperfections in annotation, the functionalities attributed to MLT2B3 in various studies may require reassessment. For example, an MLT2B3 LTR was reported to be the dominant promoter for human beta1,3-galactosyltransferase 5 in the colon (Dunn *et al.* 2005). Yet, our analysis annotates this element as two MLT2B5 fragments, and different versions of RepeatMasker are equivocal (Fig. 6). In another study, MLT2B3 is implicated in forming the stem structure of lincRNA TCONS_00011109 (Kapusta *et al.* 2013). However, LAST and different RepeatMasker versions suggest ambiguity between MLT2B3 and MLT2B5 for this element.

These findings indicate that some annotations for MLT2B3 and MLT2B5 are ambiguous. This discrepancy underscores the need for a thorough refinement of other MLT2 consensus sequences, to accurately capture their biological roles and lineage.

## 4 Conclusion

In conclusion, our study revealed new subfamilies of MLT2 LTR elements, and established new consensus sequences. We believe that although TEs in the human genome have been well studied, there is still much room for research as annotation and classification methods have not yet been perfected, which is also shown in another study (Carey *et al.* 2021b). At the same time, we have found that many transposable elements, which are functional within the genome, are actually other types of TE due to imperfect annotations. This is a topic very much worth further in-depth study.

## Supplementary Material

vbae110_Supplementary_Data

## Data Availability

The data underlying this article are available in the article and in its online supplementary material.
