# Supplementary material for: Evolution and subfamilies of HERVL human endogenous retrovirus
Source: Bioinform Adv. 2024 Jul 30;4(1):vbae110. doi: 10.1093/bioadv/vbae110 (PMC11319637; doi:10.1093/bioadv/vbae110)
Supplement: vbae110_Supplementary_Data [file vbae110_supplementary_data.pdf]

1 Methods

1.1 Analysis of Interspersed Repeats in the Human Genome

We first downloaded consensus DNA sequences of human repeat elements from Dfam (https://dfam.org/home) version 3.7, a comprehensive database of transposable element families. The human genome (GRCh38/hg38) was used as the reference genome for this study.

```
dfam-fasta -c9606 -x'root;Interspersed_Repeat' > reps.fa
```

2 Annotation of Interspersed Repeats

To annotate repeats in the human genome, we employed LAST version 1411 (https://gitlab.com/mcfrith/last). We prepared a database for the repeat sequences file 'repeats.fa' using the LAST command lastdb. This creates an indexed database named 'db' from the 'repeats.fa' file containing consensus sequences of repeats.

```
$ lastdb db repeats.fa
```

2.1 Parameter Training

Next, we used last-train (https://gitlab.com/mcfrith/last/-/blob/main/doc/last-train.rst) to determine the average rates of insertion, deletion, and substitutions between the genome and repeat consensus sequences.

```
$ last-train -P8 --revsym -X1 -m100 db human_genome.fa > rep.train
```

Table 1: Parameters Used in last-train

Parameter	Description
-P8	Utilizes eight parallel threads for faster processing
--revsym	Enforces reverse-complement symmetry of the substitution rates
-X1	Address ambiguous bases in the consensus sequences, treating N bases as ambiguous.
-m100	Increases sensitivity by allowing up to 100 initial matches per query position

2.2 Alignment

We used lastal to align the human genome sequences against the repeat database.

```
$ lastal -P8 -p rep.train -m100 -D1e7 --split db
human_genome.fa | last-postmask > genome-to-reps.maf
```

Table 2: Parameters Used in lastal

Parameter	Description
`-P8`	Accelerates the process with eight threads
`-p rep.train`	Applies pre-computed alignment parameters from 'rep.train'
`-m100`	Sets high sensitivity for alignment detection
`-D1e7`	Selects alignments that are rarely expected by chance
`--split`	Resolves ambiguity by segmenting the genome

2.3 Hybrid TE Identification

Finally, we used TE-reX (https://github.com/mcfrith/te-rex) to identify and extract "hybrid TEs" from the genome.

```
$ te-rex genome-to-reps.maf reps-to-reps.tab my-out
```

The 'reps-to-reps.tab' file has pairwise alignments between repeat consensus sequences, indicating which coordinates in one consensus are homologous to which coordinates in another.

3 Classification of Subfamilies

To refine the classification of subfamilies, we began with self-comparisons of targeted subfamily instances using `rmblast.pl` in RepeatModeler version 2.0.3 (https://github.com/Dfam-consortium/RepeatModeler) using the `-ms 2000` command to set a high minimum score threshold. The highest-scoring instance from this comparison was selected as the consensus sequence. This consensus sequence was then input into `alignAndCallConsensus.pl`.

```
$ rmblast.pl -ms 2000 -masklevel 101
```

The results from `alignAndCallConsensus.pl` were visualized using the `-html` option and stored in the `con.ali` file.

```
$ alignAndCallConsensus.pl -c con.fa -e rma2.fa -ma 14 -html
```

The `GrepCrossmatch` tool was used to identify sequences with similar features from the `con.ali` file. If a significant number of sequences differed from the consensus and shared distinct features, indicating potential new subfamilies, they were extracted using `getSeqfromXMlines.pl` for the establishment of new subfamilies.

```
$ GrepCrossmatch con.out
```

The above processes were repeated until no new subfamilies were detected. Finally, `rmblast.pl` was used again to perform self-comparisons on the newly established subfamilies, with command `-ms 2000 -masklevel 101` to ensure high minimum score, and to return all matches over the cutoff score, not only the best. This step was to ensure the differences between subfamilies.

4 Details of results

```
consensus  295 ----------TT-T-GGACT-------C-TTG----GA-C-T---T-------------------A-----C----AC--C---A-G-TGG--TTTG-C   326
aba76      290 ----------TT-T-GGACT-------C-TTG----GA-C-T---T-------------------G-----G----AC--C---A-G-TG---TTTG-C   320 [414]
aba77      290 ----------TT-T-GGACG-------C-TTG----GA-C-T---T-------------------A-----C----AC--G---G-G-CGG--TTTG-C   321 [415]
aba78      294 ----------TT-T-GGACG-------C-TTG----CA-C-T---T-------------------A-----C----AC--C---A-G-TGG--TTTG-C   325 [416]
aba79      292 ----------TT-T-GAACT-------C-TTG----GA-C-T---T-------------------A-----T----AC--C---A-G-TGG--TTTG-C   323 [417]
aba8       305 ----------TT-T-GGACT-------C-TTG----GA-T-A---T-------------------A-----C----AC--C---A-G-TGG--TTTG-T   336 [418]
aba80      293 ----------TT-T-GGACT-------C-TTG----GC-C-T---T-------------------A-----T----AC--A---A-G-GGA--TTTG-T   324 [419]
aba81      288 ----------TC-T-GGACT-------C-TTG----GA-C-C---C-------------------A-----C----AT--C---A-G-TGG--TTTT-C   319 [420]
aba82      285 ----------TT-T-TGACT-------C-TTG----GA-C-C---T-------------------A-----C----AC--C---A-G-TGG--TTTG-C   316 [421]
aba83      294 ----------TC-T-GGACC-------C-TTG----GA-C-A---T-------------------A-----T----AC--C---A-G-TGG--TTTG-C   325 [422]
aba84      290 ----------TT-T-GGACT-------C-TTG----GA-C-C---T-------------------A-----C----AC--C---A-G-TGG--TTTG-C   321 [423]
aba85      291 ----------TT-T-GGACT-------C-TTG----GA-T-T---C-------------------A-----C----AG--C---A-G-TGG--TTTG-C   322 [424]
aba86      280 ----------TT-T-GGACT-------C-TTG----AA-C-T---T-------------------A-----T----GT--C---A-G-TGG--TTTA-C   311 [425]
aba87      295 ----------TT-T-GGACT-------C-TTG----GA-C-T---T-------------------A-----G----AC--C---A-G-TGG--TTTG-C   326 [426]
aba88      283 ----------TT-T-GAACT-------C-TTG----GA-C-T---T-------------------A-----C----AC--C---A-G-TGA--TTTG-C   314 [465]
aba14      296 ----------TT-G-GGACT-------C-TTG----GA-C                                                                310 [43]
aba148     299 ----------TT-A-GGACT-------C-TTG----GA-C                                                                313 [52]
aba189     286 ----------TT-G-GGACT-------C-TTG----GA-C                                                                300 [94]
aba190     294 ----------TT-A-GGACT-------C--TG----GA-C                                                                307 [96]
aba191     296 ----------TT-G-AGACT-------C-TTG----AA-C                                                                310 [97]
aba217     294 ----------TT-G-GAACT-------C-TTG----GA-C                                                                308 [123]
aba219     295 ----------TT-G-GGACT-------C-TTG----GA-C                                                                309 [125]
aba260     308 ----------TT-G-GTACT-------C-TTG----GA-C                                                                322 [166]
aba289     292 ----------TT-G-GGATT-------C-TTG----GA-C                                                                306 [196]
aba305     287 ----------TT-G-GGACT-------C-TTG----GA-C                                                                301 [452]
aba313     295 ----------TT-T-GGACT-------C-TTG----GA-C                                                                309 [220]
aba355     286 ----------TT-G-GGACT-------C-TTA----AA-C                                                                300 [264]
aba367     290 ----------TT-T-GGGCT-------C-TCG----GA-C                                                                304 [469]
aba377     293 ----------TT-G-GGACT-------C-TTG----GA-C                                                                307 [286]
aba402     296 ----------TT-G-GGACT-------C-TTG----GA-C                                                                310 [313]
aba424     295 ----------TT-G-GGACT-------C-TTG----GA-C                                                                309 [336]
aba443     294 ----------CT-A-GGACT-------C-TTG----GA-C                                                                308 [356]
```

Figure S1: Detailed Multiple Sequence Alignment (MSA) Results of aba group. A distinct subgroup is highlighted within the red box, indicating sequences that maintain unique patterns. This subgroup exhibits consistent, characteristic difference compared to the rest of the sequences, supporting the identification of new subfamilies.

```
consensus 394 CGGACT------------------------GA--GCCAC---GCTACC--------------------G-------------------GCT---------    416

b72       427 TGGACT------------------------GA--GCCTC---GCTACT--------------------G-------------------GCT---------    449 [113]
b73       424 TGGACT------------------------GA--GTCA-----CTACC--------------------A-------------------CCT---------    444 [108]
b74       391 TGGACT------------------------GA--GGCAT---GCTACG--------------------G-------------------GCT---------    413 [60]
b75       378 CAGAAT------------------------GA--GCCGA---GCTATG--------------------G-------------------GTT---------    400 [129]
b76       408 TGGACTGAGCCTCTTTGG------------GA--GCCAC---ACTACT--------------------A-------------------G-T---------    441 [62]
b77       422 TGGACC------------------------GA--GCCAC---GCTCCC--------------------G-------------------GCT---------    444 [63]
b78       419 CAGACT------------------------GA--GCCA-----CTACT--------------------G-------------------GCT---------    439 [64]
b79       420 TGGACT-----------------------------CCA-----CTACC--------------------A-------------------GTT---------    437 [65]
b80       425 TGGACT------------------------GA--GCAGT---GCTACC--------------------A-------------------GCT---------    447 [114]
b81       417 TGGACT------------------------GA--GCCAT---GTCACT--------------------G-------------------GCT---------    439 [92]
b82       400 CAGACT------------------------AA--GCCA--------------------------------------------------GCT---------    414 [68]
b83       423 TGGACT------------------------GA--GCCA-----CTACC--------------------A-------------------ACT---------    443 [102]
b84       452 CAGACT------------------------GA--GCCA-----CTACC--------------------G-------------------GCT---------    472 [126]
b85       424 TGTACT------------------------GA--GCCA-----CTACC--------------------A-------------------GCT---------    444 [103]
b86       427 CAGACT------------------------GA--GCCA-----CTACC--------------------A-------------------GCT---------    447 [124]
b87       427 TGGATT------------------------GA--TCCAT---ACTAAT--------------------A-------------------CCT---------    449 [93]
b9        420 CGGACT------------------------GA--GCCA-----CTACT--------------------G-------------------ACT---------    440 [74]
b10       408 CAGCCT------------------------GA--GCCA                                                                 419 [1]
b12       418 TTGACT------------------------GA--GCCA                                                                 429 [94]
b13       406 TGGACT------------------------GA--GCCA                                                                 417 [115]
b22       423 TGGAGT------------------------GA--GCCA                                                                 434 [117]
b24       428 TGGACT------------------------GA--GCCA                                                                 439 [98]
b27       408 CGGACT------------------------GA--GCCA                                                                 419 [17]
b28       418 TAGACT------------------------GA--GCCA                                                                 429 [110]
b3        418 CAGACT------------------------GA--GCCA                                                                 429 [20]
b35       427 TGGAAT------------------------GA--GCCA                                                                 438 [23]
b38       417 TGGACT------------------------GA--GCCA                                                                 428 [26]
b39       413 TGGACT------------------------GA--GCCA                                                                 424 [27]
b57       415 CGGACT------------------------AA--GCCA                                                                 426 [86]
b71       383 CGGACT------------------------GA--GCCA                                                                 394 [125]
b8        410 CAGACT------------------------CA--GCCA                                                                 421 [66]
```

Figure S2: Detailed Multiple Sequence Alignment (MSA) Results of MLT2B3 group. A distinct subgroup is highlighted within the red box, indicating sequences that maintain unique patterns. This subgroup exhibits consistent, characteristic difference compared to the rest of the sequences, supporting the identification of new subfamilies.

Substitution and Indel Scores

The substitution rates form a 4x4 matrix is presented below (Table S1). These scores were used in the alignment process to ensure greater accuracy in identifying hybrid elements.

Here, `-a 25` is the deletion open cost, `-A 29` is the insertion open cost, `-b 1` is the deletion extension cost, and `-B 1` is the insertion extension cost. These rates of substitution, deletion, and insertion were used to calculate alignment probabilities, and the most likely division of the genome into parts.

Substitution and Indel Probabilities

The substitution probabilities and indel probabilities that were used to derive the above scores are shown below (Table S2). The substitution probabilities reflect the likelihood of each nucleotide being substituted for another, while the indel probabilities indicate the chances of opening and extending insertions and deletions. last-train finds probabilities (rates) and converts them to scores.

```
#last -a 25
#last -A 29
#last -b 1
#last -B 1
     A    C    G    T
A    4   -6   -2   -7
C   -7    5   -7    0
G    0   -7    5   -7
T   -7   -2   -6    4
```

Table S1: Final substitution and indel scores used in the alignment process. In the 4x4 matrix, each column corresponds to a base in the genome, and each row corresponds to a base in a repeat consensus sequence.

```
# Substitution probabilities:
#   A           C           G           T
A 0.191536    0.0148696   0.0339703   0.0215822
C 0.0200974   0.133956    0.0121832   0.0718054
G 0.0718054   0.0121832   0.133956    0.0200974
T 0.0215822   0.0339703   0.0148696   0.191536

# Indel probabilities:
# delOpenProb: 0.0305061
# insOpenProb: 0.0122328
# delExtendProb: 0.802742
# insExtendProb: 0.802567
```

Table S2: Substitution and indel probabilities used in the alignment process. In the 4x4 matrix, each column corresponds to a base in the genome, and each row corresponds to a base in a repeat consensus sequence.
